# Certolizumab pegol ınduced paradoxical psoriatic alopecia with therapeutic response to bimekizumab^؆^

**DOI:** 10.1016/j.abd.2026.501320

**Published:** 2026-03-26

**Authors:** Gamze Taş Aygar, Furkan Keleş, Feyzanur Elif Biber, Unsal Han, Selda Pelin Kartal

**Affiliations:** a Department of Dermatology, Ankara Etlik City Hospital, Ankara, Turkey; b Department of Pathology, Ankara Etlik City Hospital, Ankara, Turkey

Dear Editor,

The increasing prevalence of immune-mediated diseases has led to widespread use of biologics such as TNF-α inhibitors.^1^ A known complication of these agents is Paradoxical Adverse Reactions (PARs), often manifesting as new-onset or worsened psoriasis. Clinically and histologically, these lesions typically mimic conventional psoriasis. Improvement upon drug discontinuation and worsening with continued therapy are characteristic.^2^ We present a rare case of paradoxical psoriatic alopecia associated with certolizumab pegol, successfully managed with bimekizumab.

A 29-year-old woman with ankylosing spondylitis, treated with certolizumab pegol for 1.5-years, presented with sudden-onset scalp hair loss and erythematous, scaly plaques. There was no mucosal, nail, or other cutaneous involvement, and she had no personal or family history of dermatologic disease. Laboratory findings were within normal limits. A punch biopsy from a scalp plaque revealed parakeratotic crusting with nuclear debris, superficial hemorrhage in the stratum corneum, focal acanthosis, and loss of the granular layer, along with mild spongiosis, interface vacuolar degeneration, and dyskeratosis (Fig. 1A–B), while PAS staining was negative. This clinicopathologic constellation was consistent with TNF-α inhibitor – related paradoxical psoriatic alopecia. Certolizumab was discontinued, and bimekizumab was initiated. During follow-up, partial hair regrowth with reduced scaling of the alopecic plaque was noted at week-4. By week-8, further regrowth and near-complete resolution were observed. At the 6-month follow-up, complete resolution of the scalp scaling and restoration of normal skin texture were achieved, with near-complete hair regrowth except for mild residual thinning along the midline (Fig. 2A‒C).

**Fig. 1:**
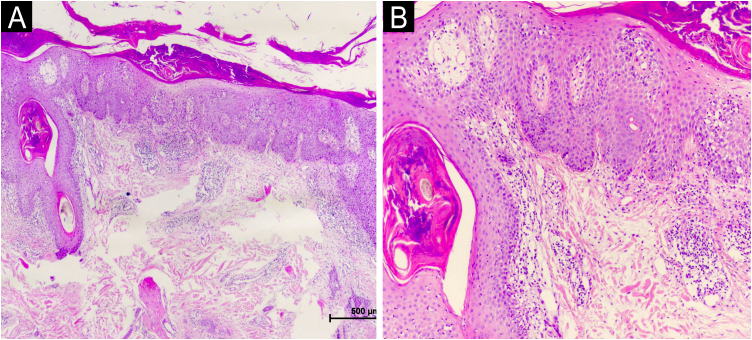
(A‒B) Histopathological findings from two punch biopsies showed similar changes: parakeratotic crust with nuclear debris and surface hemorrhage, partial acanthosis with loss of the granular layer, mild spongiosis, interface vacuolar degeneration, and isolated dyskeratosis, particularly around the follicles. In the dermis, perivascular lymphocytic inflammation was also noted.

**Fig. 2:**
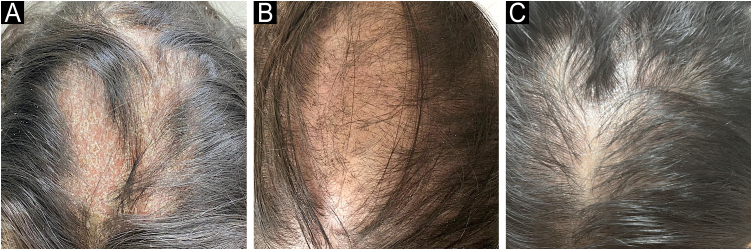
(A) Hyperkeratotic alopecic plaques with scaling and erythema located in the frontoparietal area of the scalp at presentation. (B) Further hair regrowth and near-complete resolution of the alopecic plaque after 8-weeks of bimekizumab (320 mg) therapy. (C) Complete resolution of scalp scaling and restoration of normal skin texture at the 6-month follow-up, with near-complete hair regrowth except for mild residual thinning along the midline.

Paradoxical psoriatic alopecia is an uncommon manifestation of anti-TNF therapy. Although certolizumab pegol’s unique structure ‒ a pegylated Fab fragment lacking the Fc region ‒ was expected to reduce immunogenicity, several cases of alopecia have been reported (Table 1).^3^, ^4^, ^5^, ^6^ The incidence of paradoxical psoriasis during TNF-α inhibitor therapy is around 5%, with a female predominance (60%–70%). Infliximab is most frequently implicated, followed by adalimumab, etanercept, certolizumab, and golimumab.^2^ Onset can range from weeks to years after therapy initiation;^2^ in our case, the reaction developed after 18-months of certolizumab exposure, consistent with this variability.

**Table 1:** Certolizumab-ınduced paradoxical psoriatic alopecia – case reports ordered by year.

Author (year)	Underlying disease	Onset of alopecia (months)	Pre-treatment clinical features	Intervention / Treatment	Outcome
Miguel et al. (2019) ^3^	Sacroiliitis	6	Hyperkeratotic plaques, alopecia; biopsy showing AA-like features and psoriasiform hyperplasia	Discontinuation of certolizumab, initiation of ustekinumab	Complete hair regrowth in 16-weeks
Ferraresso et al. (2020) ^4^	Crohn’s disease	1	Psoriasiform scalp lesions, hair loss	Discontinuation of certolizumab, topical clobetasol	Regression of hair loss, clinical improvement
Gawdzik et al. (2020) ^5^	Ankylosing spondylitis	–	Palmoplantar pustulosis, PG-like lesions, alopecia of the scalp	Discontinuation of certolizumab, secukinumab + methotrexate	Clinical improvement
Lauro et al. (2023) ^6^	Psoriasis	10	Psoriatic alopecia of the scalp	Discontinuation of certolizumab, oral dexamethasone + topical clobetasol	Clinical resolution
Present Case (2025)	Ankylosing spondylitis	18	Erythematous, scaly plaques, alopecia	Discontinuation of certolizumab, initiation of bimekizumab	Complete hair regrowth at week-8

Histopathological findings in biologic-induced paradoxical psoriatic alopecia often demonstrate a psoriasiform pattern; however, increased dermal eosinophils, interface changes, and dyskeratosis have been reported as distinguishing features from classic psoriasis.^7^ In our case, parakeratosis and loss of the granular layer were accompanied by marked interface degeneration and dyskeratosis, supporting the diagnosis of a TNF-α inhibitor-related paradoxical reaction. Recognition of these histopathological differences may provide valuable clues in the differential diagnosis and guide more tailored therapeutic strategies.

The key mechanism involves excessive IFN-α production by plasmacytoid dendritic cells, promoting Th17 polarization and increased IL-17/IL-22, which activate keratinocytes and sustain inflammation. The presence of Th17 cells and IL-17 in affected lesions, along with good response to secukinumab, supports IL-17 inhibitors as viable treatment options.^8^

Treatment selection for TNF-induced PARs depends on severity and comorbid conditions. Given the dual inhibition of IL-17A and IL-17 F by bimekizumab, and its proven efficacy in both psoriasis and ankylosing spondylitis, it was chosen in this case.^9^ While data on its use in psoriatic alopecia are limited, our case aligns with emerging evidence favoring IL-17 – targeted therapy in managing TNF inhibitor – induced dermatologic reactions.

Although paradoxical reactions to IL-17 inhibitors, including bimekizumab, have also been reported,^10^ to our knowledge, this is the first published case of certolizumab pegol – induced psoriatic alopecia successfully treated with bimekizumab. Further studies are needed to clarify its long-term safety and efficacy in this rare condition.

## ORCID ID

Furkan Keleş: 0009-0002-9187-7301

Feyzanur Elif Biber: 0009-0007-5872-6241

Unsal Han: 0000-0003-1952-4499

Selda Pelin Kartal: 0000-0001-7310-8635

## Financial support

The authors received no financial support for this study.

## Authors' contributions

Gamze Taş Aygar: Approval of the final version of the manuscript; critical literature review; data collection, analysis and interpretation; effective participation in research orientation; ıntellectual participation in propaedeutic and/or therapeutic management of studied cases; preparation and writing of the manuscript.

Furkan Keleş: Approval of the final version of the manuscript; critical literature review; data collection, analysis and interpretation; effective participation in research orientation; preparation and writing of the manuscript.

Selda Pelin Kartal: Approval of the final version of the manuscript; ıntellectual participation in propaedeutic and/or therapeutic management of studied cases; manuscript critical review.

Feyzanur Elif Biber: Approval of the final version of the manuscript; ıntellectual participation in propaedeutic and/or therapeutic management of studied cases.

Unsal Han: Approval of the final version of the manuscript; Intellectual participation in propaedeutic and/or therapeutic management of studied cases.

## Research data availability

Does not apply.

## Conflicts of interest

None declared.
